# Multi-state models and arthroplasty histories after unilateral total hip arthroplasties: Introducing the Summary Notation for Arthroplasty Histories

**DOI:** 10.3109/17453674.2012.684140

**Published:** 2012-06-04

**Authors:** Marianne H Gillam, Philip Ryan, Amy Salter, Stephen E Graves

**Affiliations:** ^1^School of Population Health and Clinical Practice; ^2^Data Management and Analysis Centre, University of Adelaide; ^3^Australian Orthopaedic Association National Joint Replacement Registry, Adelaide, Australia.; Correspondence : marianne.gillam@adelaide.edu.au

## Abstract

**Background and purpose:**

An increasing number of patients have several joint replacement procedures during their lifetime. We investigated the use and suitability of multi-state model techniques in providing a more comprehensive analysis and description of complex arthroplasty histories held in arthroplasty registries than are allowed for with traditional survival methods.

**Patients and methods:**

We obtained data from the Australian Orthopaedic Association National Joint Replacement Registry on patients (n = 84,759) who had undergone a total hip arthroplasty for osteoarthritis in the period 2002–2008. We set up a multi-state model where patients were followed from their first recorded arthroplasty to several possible states: revision of first arthroplasty, either a hip or knee as second arthroplasty, revision of the second arthroplasty, and death. The Summary Notation for Arthroplasty Histories (SNAH) was developed in order to help to manage and analyze this type of data.

**Results:**

At the end of the study period, 12% of the 84,759 patients had received a second hip, 3 times as many as had received a knee. The estimated probabilities of having received a second arthroplasty decreased with age. Males had a lower transition rate for receiving a second arthroplasty, but a higher mortality rate.

**Interpretation:**

Multi-state models in combination with SNAH codes are well suited to the management and analysis of arthroplasty registry data on patients who experience multiple joint procedures over time. We found differences in the progression of joint replacement procedures after the initial total hip arthroplasty regarding type of joint, age, and sex.

Since its inception in 1999, the Australian Orthopaedic Association National Joint Replacement Registry (AOANJRR) has collected data on more than 650,000 joint replacement procedures, and it currently captures almost 100% of all primary and revision joint replacements performed in Australia (AOANJRR 2010).

Arthroplasty registry data are conventionally analyzed using survival methods where the outcome is time to one event of interest, which is usually the time from the primary procedure until revision of the prosthesis. Other outcomes may also be of interest, for example, time to death or to receiving another arthroplasty, as well as the association between covariates and these events. In addition, the rise in life expectancy of the population combined with an increasing number of joint replacements being performed has resulted in many patients experiencing several joint replacement procedures during their lifetime. Thus, the arthroplasty history of patients may eventually become rather complex. For example, a patient may undergo one primary arthroplasty, then a second followed by a revision of the first arthroplasty, and then a third arthroplasty or another revision and so on. There is a need for statistical methods that are able to describe and analyze the more complex arthroplasty history data that are collected by joint registries. At the same time, there is a need to specify which of the patient’s joints have had an arthroplasty or revision and the order of events at any specific point in time.

Multi-state models, which are a generalization of traditional survival models, allow for a detailed description of this event history. They model processes whereby the individual occupies and moves between a finite number of states. The states describe conditions, such as having had a joint replacement or having had a revision. A transition, or an event, occurs when an individual changes state (Hougaard 1999). There are 2 types of states: absorbing, if no transition out of it is allowed (e.g. being dead), or transient, when a subject can experience further events such as a second arthroplasty following a revision of the first arthroplasty. An example of a simple multi-state model is the competing-risks model, where the subject can move from the initial state—for example, a joint replacement—to one of several possibly absorbing states such as being dead or revision (revision being absorbing if it is the primary endpoint of the analysis) (Gillam et al. 2010). In more complex multi-state models, individuals can move into 1 or more transient states before reaching an absorbing state, e.g. being dead. Once the state structure of the multi-state model is specified, the model can provide probabilities and hazard rates associated with states and with movements from one state to another (Andersen and Pohar Perme 2008).

We hypothesized that multi-state models would be well suited for analysis of data on complex arthroplasty histories held in arthroplasty registries. In order to enable functional multi-state modeling and to provide a shorthand method of recording and communicating patient-level arthroplasty histories, we developed the Summary Notation for Arthroplasty Histories (SNAH), which will be presented in this paper. Data from the AOANJRR were analyzed using a multi-state model to describe numbers and types of arthroplasty procedures, to estimate state occupation probabilities and the effect of sex on transition hazards between states in a cohort of patients who received total hip arthroplasties for osteoarthritis.

## Material and methods

Data on patients who received a unilateral total hip arthroplasty for osteoarthritis in the period from January 1, 2002 to December 31, 2008 were obtained from the AOANJRR. Patients aged 55–84 years were selected in order to keep the data homogeneous, and for descriptive purposes they were categorized into 3 groups based on age (55–64, 65–74, and 75–84 years).

### Summary Notation for Arthroplasty Histories

We developed the Summary Notation for Arthroplasty Histories (SNAH) to facilitate the description and analysis of joint replacement event history data. In the SNAH code, a patient’s arthroplasty history is summarized as an alphanumeric string. The string is composed of 4-character elements, with each element, or event, representing an arthroplasty. Events are separated by a special character (usually a forward slash) and may be concatenated in order of time to form the arthroplasty history, which can be updated as new events are recorded. The generic form of the event is: ‘JSnm’ where ‘J’ represents the anatomical location of the arthroplasty, ‘S’ represents the side (right or left), ‘n’ represents the cumulative number of arthroplasties to date, and ‘m’ represents the cumulative number of revisions of joint ‘J’. In each event, ‘n’ permits interpretation of an individual event if the history becomes disaggregated, and allows reconstruction of the time sequence of events if the full arthroplasty history becomes corrupted. For example, ‘HR10’ denotes that the first ever arthroplasty was of the right hip (and that no revisions of this arthroplasty have yet occurred); ‘HR21’ denotes that the second arthroplasty was revision of the right hip. The SNAH code KR10/KL20/HR30/KL41/KL52/ describes a patient who has had 5 joint procedures: a primary right knee, then a primary left knee, then a primary right hip, then a revision of the left knee followed by a second revision of the left knee. This coding allows for an easy summary of patients’ arthroplasty histories in addition to enabling management and analysis of data with multiple events. The notation is described in detail in the Appendix (see Supplementary data (www.actaorthop.org), identification number 5260).

### Multi-state modeling

**Figure 1.**
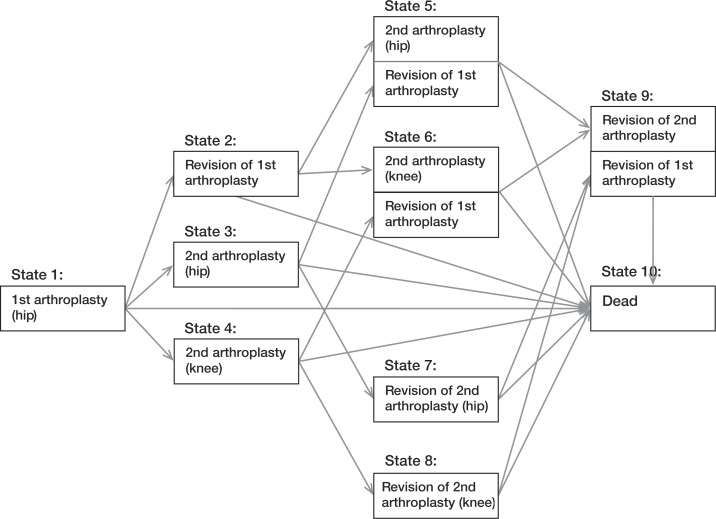
Multi-state model with 10 states for patients who received a first hip arthroplasty possibly followed by a second arthroplasty (hip or knee), revisions of these, and death.

We developed a multi-state model for a restricted scenario in which patients were followed from their first recorded arthroplasty to several possible transient states: revision of the first arthroplasty, a second arthroplasty (hip or knee), revision of the second arthroplasty and the absorbing state, dead (we adopt the naming convention that ‘death’ is an event and ‘being dead’ is a state (Hougaard 1999)). The model, with 10 possible states that can be occupied (boxes) and paths that can be travelled (arrows), is illustrated in Figure 1. Based on this model , we calculated the numbers and proportions of patients in the cohort who experienced each event during the study period. For illustrative purposes, our model is simple; it only relates to 2 primary arthroplasties, first revisions of these, and death. The multi-state method can be extended to include further possible events, such as a third or fourth arthroplasty with associated revisions and re-revisions, but we do not do so here.

State occupation probabilities, the estimated proportions of patients in a given state at a given time after the initial hip arthroplasty, were calculated using the Aalen-Johansen estimator (Aalen and Johansen 1978). We assumed that the censoring was independent of the states occupied and the transition times (Datta and Satten 2001).

We used a Cox proportional hazards model (Cox 1972) to estimate the effect of sex, adjusted for age, on the transition hazards between states—that is, the instantaneous risk of a subject moving from one state to another at a given point in time conditional on being at risk for that particular transition. A preliminary analysis indicated that time spent in the current state, but not in the previous state, affected the transition hazards; hence, we chose a model where time was reset (clock-reset model or semi-Markov (Putter et al. 2007)) after entering a new state. The Cox model was stratified on transitions such that transition hazards were calculated for each possible transition and the covariates (age and sex) were transition-specific. The proportional hazards assumption in the Cox model was checked with Schoenfeld residuals for each transition.

Observations were right-censored on December 31, 2008 after the last event (either first arthroplasty, second arthroplasty, or revisions of first and/or second arthroplasty) if death had not yet occurred. Revisions are re-operations of previous hip or knee replacements where one or more of the prosthetic components are replaced or removed, or another component is added (AOANJRR 2010).

For the analyses, we used the ‘mstate’ package (de Wreede et al. 2010) in the software environment ‘R’ (R Development Core Team 2011).

## Results

The distribution at the start of the study period of 84,759 patients in the 3 age groups was 22,885 (27%) in the 55- to 64-year group, 34,833 (41%) in the 65- to 74-year group, and 27,041 (32%) in the 75- to 84-year group. If patients received a second arthroplasty, it was usually a total hip prosthesis or a total knee prosthesis, but a few patients received a partial hip prosthesis or unicompartmental knee prosthesis.

In this description of the results, the term ‘events’ refers only to the type of events covered in this multi-state model, i.e. first primary arthroplasty (hip), second primary arthroplasty (either hip or knee), first revisions of these, and death.

Table 1 shows the numbers and proportions of arthroplasty events that had occurred at the end of the study period. Following the first hip replacement (either left or right), 3 times as many patients had a contralateral primary hip replacement as a left or right knee replacement (12% vs. 4%), 2% had a revision, and 5% died (Figure 1: from state 1 to 2, 3, or 4). Of the 1,929 patients who had a revision after the first arthroplasty, 6% subsequently received a hip and 4% received a knee (Figure 1: from state 2 to 5 or 6). Of patients who had received a second arthroplasty, either hip (9,997) or knee (3,565), 1% went on to have a revision of the first arthroplasty and 2% went on to have a revision of the second arthroplasty (Figure 1: from state 3 to 5 or 7 and from state 4 to 6 or 8).

**Table 1.** Numbers and percentages of events in the 10-state model (Figure 1) at the end of the study period for patients whose first arthroplasty was either a left total hip arthroplasty for osteoarthritis or a right total hip arthroplasty for osteoarthritis

To:		2	3	4	5	6	7	8	9	10	No further event **^a^**	Total entering
From:												
1:	1st arthroplasty	1,929	9,997	3,565						4,365	64,903	84,759
	hip	2%	12%	4%						5%	77%	100%
2:	Revision of 1st				107	80				141	1,601	1,929
	arthroplasty				6%	4%				7%	83%	100%
3:	2nd arthroplasty				79		221			345	9,352	9,997
	hip				1%		2%			3%	94%	100%
4:	2nd arthroplasty					38		85		110	3,332	3,565
	knee					1%		2%		3%	93%	100%
5:	2nd arthroplasty hip								5	7	174	186
	/revision of 1st hip								3%	4%	94%	100%
6:	2nd arthroplasty knee								1	3	114	118
	/revision of 1st hip								1%	3%	97%	100%
7:	Revision of 2nd								6	10	206	221
	arthroplasty hip								2%	5%	93%	100%
8:	Revision of 2nd								3	4	79	85
	arthroplasty								2%	5%	93%	100%
9:	Revision of 1st or									0	13	13
	2nd arthroplasty									0%	100%	100%
10:	Dead											

**^a^** Refers to the number of patients who entered the state and had not experienced any further events covered in this multi-state model at the end of the study.

**Figure 2.**
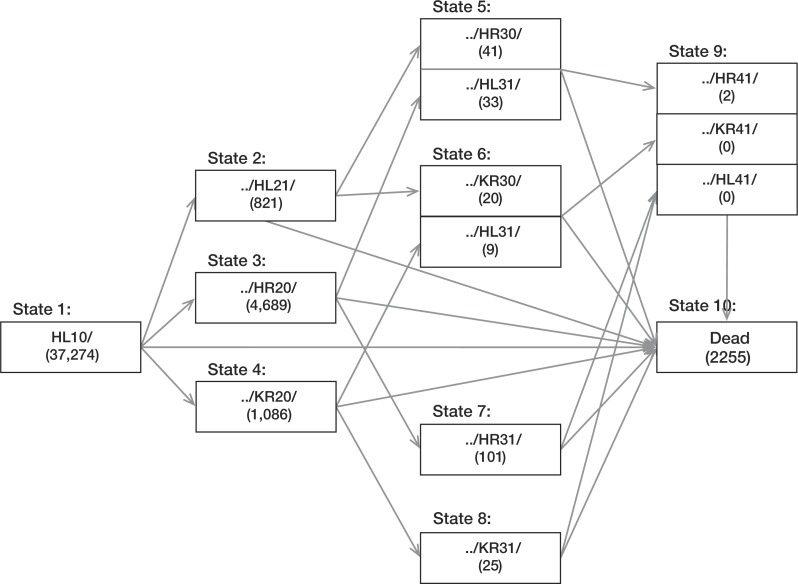
Example of the multi-state model with SNAH code on a subsample of patients who received a left hip prosthesis as first arthroplasty, followed by another primary arthroplasty or a revision of the left hip. (Number of events is shown in parentheses).

Figure 2 shows an example of the multi-state model with the SNAH code on a subsample of patients who received a left hip as first arthroplasty. (We arbitrarily chose to show the number of subsequent right-knee primary arthroplasties after the first hip, but one could instead have chosen left knees or both left and right knees). State 6, for example, consists of 20 patients with histories of HL10/HL21/KR30/ and 9 patients with HL10/KR20/HL31/.

**Figure 3.**
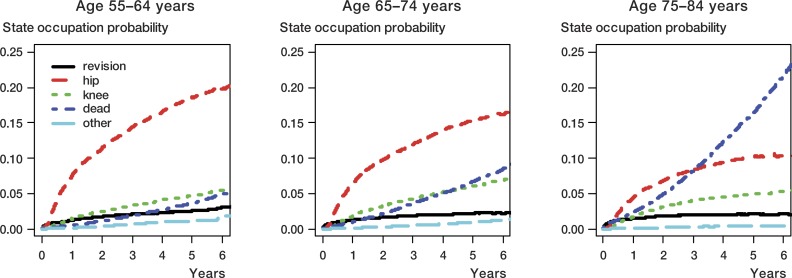
State occupation probabilities for patients in 3 age groups after first hip arthroplasty, based on the model in Figure 1 (revision: state 2; hip: state 3; knee: state 4; dead: state 10; other: states 5–9).

The estimated state occupation probabilities at different time points since the first recorded hip arthroplasty for each age group are presented in Figure 3. Because so few patients experienced events beyond the second event after the initial arthroplasty (Table 1, states 5–9), these events were combined. Among patients in the 2 youngest age groups, it appears that at each time point after having received the first hip arthroplasty the probability of occupying state 3 (having received a second hip arthroplasty and no further events) was higher than occupying any of the other event states. For the oldest age group, the probability of occupying state 3 was the highest until approximately 3 years after the first hip prosthesis; thereafter, the probability of being in the state ‘dead’ was the highest. The probability of occupying state 3 decreased with increasing age. For example, 5 years after having received a first total hip arthroplasty approximately 20% of patients aged 65–74 years were estimated to have received a contralateral hip (and had not experienced any other event in this model) as compared to 10% of patients in the oldest age group (aged 75–84 years). The estimated probability of occupying state 4 (having received a knee arthroplasty) rather than occupying state 3 was much lower throughout the study period. Thus, when a patient received a hip first, this was more likely to be followed by another hip than a knee.

Table 2 shows the effect of gender on the transition hazards between the states pictured in Figure 1 for the 3 age groups. Because so few patients experienced events beyond a second event, these transitions are not included in the table. The transition rate from state 1 to 2 (from first arthroplasty to revision of the same arthroplasty) was higher for males than for females in the oldest (75- to 84-year) age group (HR = 1.3, 95%CI: 1.1–1.5; p = 0.004), but there was no evidence of a difference between the sexes for the youngest age groups for this transition. For the transition rates of receiving a second arthroplasty (hip or knee), these were lower for males than for females in all age groups (HR = 0.8–0.9; p < 0.05). The instantaneous risk of dying following the first arthroplasty was higher for males than for females in all 3 age groups (HR = 1.5, 1.6, and 1.7, respectively; all p < 0.01) during the study period. For patients in the oldest age group, the transition hazards to being dead from receiving second hip or receiving a knee were also higher for males than for females (HR = 2.1 and HR = 1.9, respectively; p < 0.01). In summary, the transition rates for revision after first arthroplasty were higher for males than for females in the oldest age group. In all age groups, males had lower transition rates for receiving a second primary arthroplasty of either hip or knee. Generally, males had a higher death rate than females.

**Table 2.** Effect of sex, adjusted for age, on the transition hazards between states (see Figure 1) for patients whose first arthroplasty was a total hip arthroplasty for osteoarthritis

	Age group: 55–64 years		Age group: 65–74 years		Age group: 75–84 years	
State	HR	95% CI	HR	95% CI	HR	95% CI
1→2	0.95	0.80–1.13	1.04	0.90–1.20	1.26 **^c^**	1.07–1.48
1→3	0.93	0.87–1.001	0.86**^ a^**	0.81–0.92	0.80**^ a^**	0.73–0.87
1→4	0.80 **^a^**	0.69–0.92	0.78**^ a^**	0.71–0.86	0.85 **^b^**	0.75–0.96
1→10	1.45 **^c^**	1.20–1.75	1.59**^ a^**	1.43–1.78	1.73**^ a^**	1.61–1.88
2→5	0.63	0.35–1.14	1.33	0.71–2.52	1.33	0.57–3.08
2→6	0.80	0.34–1.90	0.72	0.39–1.35	4.80 **^b^**	1.57–14.68
2→10	2.24	0.55–9.08	1.15	0.66–2.01	1.54	1.00–2.40
3→5	1.05	0.50–2.20	0.74	0.38–1.43	3.15 **^c^**	1.03–9.64
3→7	1.27	0.81–1.99	1.18	0.78–1.77	1.08	0.61–1.92
3→10	1.21	0.70–2.09	1.71 **^b^**	1.22–2.40	2.11**^ a^**	1.54, 2.90
4→6	1.48	0.50–4.41	1.52	0.62–3.75	0.39	0.05–3.33
4→8	1.33	0.60–2.97	1.78	0.97–3.28	2.55 **^c^**	1.03–6.36
4→10	5.21 **^c^**	1.10–24.58	1.64	0.83–3.28	1.87 **^c^**	1.16–3.02

HR: hazard ratio (male/female) adjusted for age.

**^a^** p < 0.001; **^b^** p < 0.01; **^c^** p < 0.05 for comparing the effect of males and females on the transition intensity.

## Discussion

We found that the arthroplasty histories had several interesting features. At the end of the study period, 12% of patients had received a second hip—3 times as many as had received a knee. Relatively few patients had more than 2 arthroplasty procedures, but with a longer observation time the number would be expected to increase.

The estimated state occupation probabilities indicated that a randomly chosen patient from any of the 3 age groups was most likely to occupy the event state of ‘having received a second hip arthroplasty’ until approximately 3 years after the first arthroplasty. After that, the patients in the oldest age group (75–84 years) were most likely to have died. Since being dead is an absorbing state, the state occupation probability of this will continue to increase with longer observation time, whereas patients will leave the transient states and the relative pattern between states may change. Hence, the model provides information on the evolving nature of patients’ arthroplasty histories. The state occupying probability of ‘having received another hip’ was largest in the youngest age group and smallest in the oldest age group. This may suggest a decreasing propensity to have further arthroplasty procedures with increasing age. However, patients in the oldest age group were also most likely to have already received a hip arthroplasty before the start of the study period, thus not being at risk of receiving a second hip arthroplasty.

Both the descriptive statistics and the estimated state occupation probabilities indicated that patients who received a first total hip arthroplasty were more likely to receive a contralateral hip than a knee arthroplasty. In data not shown here, using the same multi-state model, we found a similar pattern in a cohort of patients who received a total knee arthroplasty as first procedure; that is, the patients were more likely to receive a contralateral knee than a hip. Total hip arthroplasty (THA) and total knee arthroplasty (TKA) are considered to be surrogates for the incidence of end-stage osteoarthritis (Shakoor et al. 2002). Shakoor et al. (2002) studied the distribution of subsequent total joint replacements in patients with osteoarthritis after the initial hip or knee arthroplasty. They found that patients who received THA or TKA for osteoarthritis were more likely to receive a second arthroplasty in the contralateral joint than in other joints, suggesting that the progress of osteoarthritis in weight-bearing joints is not random. Multi-state models could provide a useful tool for further research and insight into the pattern of developing end-stage osteoarthritis. This is the subject of one of our current studies.

The effect of gender on the transition hazards between states—that is, the instantaneous risk of experiencing the event—was lower for males than for females transferring from the first arthroplasty to the second, but higher transferring into the dead state. If the mortality rate is an indicator of frailness in a population, then of those patients who received hip arthroplasties, men were more frail than women—and this may be the reason that the hazard rate for receiving a second arthroplasty was lower for men than for women. Another explanation is that women may have more extensive severe osteoarthritis than men (Srikanth et al. 2005).

Simple survival analysis—that is, analysis of time to occurrence of one event—is one of the most commonly used methods in clinical research. Multi-state models, which are a generalization of simple survival analysis, may be applied to data where there are several events of interest per individual occurring over time. The methods have been used in analyzing bone marrow transplant studies (Klein and Shu 2002), in cancer studies (Cook and Major 2006, Putter et al. 2006, Uhry et al. 2010), and in studies on HIV (Sommen et al. 2009). There is increasing interest in application of multi-state models in medical research (de Wreede et al. 2010), and there is a rich literature on the theory (Andersen et al. 1993, Commenges 1999, Hougaard 1999, Andersen and Keiding 2002, 2011). By modeling the event histories as states that the individual can occupy and move between, the models can be used to investigate and make statistical inferences about probabilities and effects of covariates on occurrence of various events in the model (Andersen and Pohar Perme 2008), thereby providing insight into the nature of progression of disease. When the models are used to predict outcomes (e.g. in cancer or joint replacement studies), new information can be incorporated and predictions adjusted as more data on the types of events the patients have experienced and the treatment they have received become available.

Multi-state modeling offers a flexible approach to analysis of arthroplasty registry data. Our particular multi-state model could be used to investigate other scenarios, such as arthroplasty histories of patients who have received total knee replacement as the initial primary arthroplasty. Furthermore, multi-state models with more events, such as third and fourth arthroplasties, or re-revisions, could be estimated. However, the flexibility of the design may cause problems in that models may become too complex if many events are included, leading to difficulties in interpretation of the results. In addition, one must ensure that the events included in the model fit the research questions. Care must also be taken in the choice of time scale (Putter et al. 2007). When the multi-state model is Markov, the assumption is that the hazard rates are independent of the history of the process, i.e. independent of past states and time spent in the current state. If they are only dependent on time spent in the current state, it is a semi-Markov model. Statistical inference is easier when the process is Markov than non-Markov, but estimates of transition hazards and state occupation probabilities are robust in semi-Markov models as long as the censoring is independent (Datta and Satten 2001). For non-Markov processes with dependent censoring, other methods have been developed (Gunnes et al. 2007). Thus, the applicability of the models to clinical research depends to a certain extent on whether the processes are Markov or non-Markov. In our data, the time between the previous event and the current event did not affect the transition rates but the time in the current state did. We therefore used a semi-Markov model where time was reset as patients entered a new state.

With increasing data on patients with multiple arthroplasty events held in arthroplasty registries, statistical methods other than those traditionally employed for single-outcome data are required. Multi-state modeling allows a more comprehensive understanding of the data and also enables analysis from the standpoint of the entire clinical history rather than focusing on the outcome of the joint replacement procedure in isolation. We have demonstrated the usefulness and suitability of multi-state models in the description and analysis of arthroplasty registry data on patients who experience multiple joint procedures over time. The use of the model was facilitated by the SNAH code. Without this tool, management of the complex data would have been difficult. The code was developed mainly to enable multi-state modeling of arthroplasty registry data and to manage data on patients with multiple joint procedures. The notation is simple and logical, and one could imagine its use in many other contexts such as clinical histories.

## Appendix

For Supplementary data, see www.actaorthop.org, identification number 5260.
