# Supplementary material for: Multi-state models and arthroplasty histories after unilateral total hip arthroplasties: Introducing the Summary Notation for Arthroplasty Histories
Source: Acta Orthop. 2012 Jun 4;83(3):220–6. doi: 10.3109/17453674.2012.684140 (PMC3369145; doi:10.3109/17453674.2012.684140)
Supplement: Supplementary file 1 [file ORT-1745-3674-83-220-s5260.pdf]

## Supplementary article data

# Multi-state models and arthroplasty histories after unilateral total hip arthroplasties

## Introducing the Summary Notation for Arthroplasty Histories

Marianne H Gillam[1], Philip Ryan[1,2], Amy Salter[1], Stephen E Graves[3]

[1]School of Population Health and Clinical Practice and [2]Data Management and Analysis Centre, University of Adelaide; [3]Australian Orthopaedic Association National Joint Replacement Registry, Adelaide, Australia.
Correspondence : marianne.gillam@adelaide.edu.au
Submitted 11-11-25. Accepted 12-02-11

## Appendix

### *Summary Notation for Arthroplasty Histories—SNAH*

SNAH was developed to provide a shorthand method of recording and communicating patient-level arthroplasty histories for major bilateral joints. The defined structure also facilitates manipulation of events by statisticians, programmers and database administrators.

In SNAH, a patient's arthroplasty history is summarized by an alphanumeric string. The string is composed of the following elements:

**(i)** **events:** an event represents an arthroplasty, either primary or revision, or death.
**(ii)** **separation characters:** events are separated by a separation character.
**(iii)** **termination character:** this is the final character of the arthroplasty history string to date.

Events (with their separation character) are concatenated, in order of time, to form the arthroplasty history which can be updated as new events are recorded.

Elements of the event strings were chosen to avoid, wherever possible, ambiguity or potential problems with renditions on screen or printed output. So, for example, no subscripts or superscripts are allowed. Font styles and effects such as italics or bold-face are not used. Upper/lower case may be used in the first character of the event string (vide infra), and the choice of case is informative, but the adjacent second character is always upper case so that the case of the first character should always be obvious.

### *(i) An event has one of two forms, (a) or (b) below:*

**(a) JSnm**

where:

**J** represents the anatomical location of arthroplasty: {J, H, K, A, S, E, W, | j, h, k, a, s, e, w}

J, j: unspecified joint
H, h: Hip
K, k: Knee
A, a: Ankle
S, s: Shoulder
E, e: Elbow
W, w: Wrist

Lowercase rendering of the first character in the event denotes that the event is an incomplete procedure such as the first stage of a planned two-stage revision. The first character of the event representing the concluding stage of the revision reverts to upper case.

**S** represents the side: {R, L}

R: right
L: left

The notation for side is capitalized to avoid confusion in some fonts of the lowercase "l" with the numeral "1", and also to provide a visual cue for the case of the adjacent first, "J", character, which may be upper or lower case.

**n** represents the cumulative number of major arthroplasties, irrespective of J and S, including the operation described by the current event: {1,2,3,4....}. An event representing a stage of a two-stage revision is treated as any other event: n increases by 1 at each stage.

Note that the value of n in each event allows for interpretation of an individual event should the history be disaggregated, and for reconstruction of the time sequence of events should the full arthroplasty history be corrupted.

**m** represents the cumulative number of revisions on the joint described by J and S in the current event: {0,1,2,3...}. Note that every stage of a multi-stage procedure has the same value of m. m=0 denotes a primary joint replacement procedure.

*Open Access - This article is distributed under the terms of the Creative Commons Attribution Noncommercial License which permits any noncommercial use, distribution, and reproduction in any medium, provided the source is credited.*
*DOI 10.3109/17453674.2012.684140*

In fact, both n and m are hexadecimal numbers. For most patients, n = {1...9} will suffice to describe their history, but for patients with even longer histories, n = {1...9, A...F} provides for up to 15 events, while still requiring only one character. (Of course, a natural extension of this system: n = {1...9, A...Z} allows for up to 35 arthroplasties.)

or:

**(b) D** denoting death. Note that the character must be capitalized.

### *(ii) Separation characters:*

Each event is separated from its time ordered neighbour(s) by a single character which, at present, may be:

**(a) /** {forward slash} the standard separator;

or

**(b) &** {ampersand} denotes two adjacent procedures occurring on the same day, or same admission, or some otherwise defined period during which it is considered the procedures happen simultaneously.

### *(iii) Termination character:*

At present there are two possible termination characters:

**(a) /** {forward slash} the standard separator is also the usual termination character

**(b) ?** {question mark} denotes that it is known the arthroplasty history to date is incomplete

### *Examples of events:*

HR10 Primary right hip arthroplasty

HL20 Primary left hip; patient's second major arthroplasty

KR21 First revision on right knee; second major arthroplasty (logically the first must have been the primary on the right knee)

KR11 not possible, cannot have a revision as the first event

HR32 second revision on right hip (the first of the two preceding events must have been the primary on the right hip, the second must have been the first revision of that hip)

HR42 second revision on right hip; one of the three preceding arthroplasties must have been the primary on the right hip, another must have been the first revision, but without knowledge of other events in the history, we do not know the nature of the other arthroplasty.

hL31 first of at least two stages of a left hip revision (note use of lowercase "h"). This is the third operation overall, one of the preceding procedures would have been the primary procedure on the left hip.

KLB4 fourth revision of a left knee; this was the 11th arthroplasty of this busy patient's history (hexadecimal character B=11 in base 10).

### *Examples of Arthroplasty Histories (AH):*

HR10/KL20/HR31/HR42/ Primary right hip, then primary left knee, then two revisions of the right hip; the revisions were not staged and they were not done on the same day/ admission. Standard event separator is also used as the standard termination character after the last known procedure (2nd revision of right hip).

HR10/KR21? Check the data! [History should be HR10/ KR20/KR31/ assuming it is known that the hip primary was the first operation.] For the time being the special termination character "?" signals a problem with the string.

KR10/HR20/HL30/HR41&HL51/ Both hips revised at the same time. The time order (n = 4 and 5) of the revisions on the right and left hip might be arbitrarily assigned.

HR10/KR20/HR31/KR41/D/ First arthroplasty was a right hip followed by a right knee arthroplasty, a revision of the first hip and finally a revision of the knee. The patient is deceased.

HR20/KR30&KL40? Primary right hip, followed by primary right and left knees on same day (or same admission). Note the termination character is "?" because we do not have a record of the first arthroplasty procedure: it could be a primary left hip, but it could also be a shoulder, elbow, wrist etc...

HR10/hR21/HR31/KL40/ Right primary hip followed by two-stage revision of right hip followed by left primary knee. Note first stage of revision has lowercase "h", second (final) stage is uppercase. m=1 for both stages.

HR10/hR21/KL30/HR41/ Right primary hip followed by first stage of planned two-stage revision, then an intervening primary left knee, then completion of the two-stage right hip revision. We know that the second event was the first step in a planned two-stage revision (because the first character of the event is in lowercase), and we look along the history until we come to its concluding pair, identified by the same 1st (but now uppercase), 2nd and 4th characters.

HR10/hR21/KL30? Same as above but staged revision of right hip is incomplete. This is not necessarily a problem with the data - the second stage simply may not yet have occurred, but the special termination character is nevertheless used as an alert.
